# Spectrum mining of immune checkpoint inhibitor-related cutaneous toxicities and analysis of associated factors based on FAERS

**DOI:** 10.3389/fphar.2025.1684390

**Published:** 2026-01-02

**Authors:** Siyao Ma

**Affiliations:** Cancer Hospital of China Medical University, Liaoning Cancer Hospital and Institute, Shenyang, China

**Keywords:** immune checkpoint inhibitors, immune-related skin adverse reactions, pharmacovigilance, signal mining, correlation analysis

## Abstract

**Objective:**

The study sought to delineate the cutaneous toxicity spectrum of immune-checkpoint inhibitors by using the US FDA Adverse Event Reporting System, and to qualify association with other immune-related adverse events (irAEs).

**Methods:**

Disproportionality analyses relied on Reporting Odds Ratio (ROR), Bayesian Confidence Propagation Neural Network (BCPNN) and proportional reporting ratio (PRR). Univariable and multivariable logistic regression were then applied to identify other irAEs that predicted irAE-Cutaneous.

**Results:**

(1) Most reports involved men aged >65 years. (2) Toxicity spectrum and outcomes: Most reports involved men over 65. Five drugs showed positive signals via the three algorithms. Immune-mediated dermatitis had the strongest signals across all. Ipilimumab strongly linked to vitiligo (ROR = 45.531); nivolumab (ROR = 9.656) and pembrolizumab (ROR = 8.376) to psoriasiform dermatitis; tislelizumab to acquired epidermolysis bullosa (ROR = 8.376). Rash and pruritus were common but weakly specific. Pembrolizumab had highest RORs for toxic epidermal necrolysis (ROR = 4.384) and Stevens-Johnson syndrome (ROR = 3.599). Nivolumab had most severe event reports, then pembrolizumab. (3) Association with other irAEs: Univariable analysis linked pneumonitis, hyperthyroidism, myositis, fatigue, hypothyroidism to skin irAEs (all P < 0.05); multivariable analysis found hyperthyroidism, myositis, fatigue, hypothyroidism as independent predictors (P < 0.05).

**Conclusion:**

Distinct toxicity spectra of each drug were observed. Hyperthyroidism, myositis, fatigue and hypothyroidism independently increase the likelihood of irAE-Cutaneous.

## 1 Introduction

Immune checkpoint inhibitors (ICIs) serve as a core regimen of tumor immunotherapy, enhancing the body’s anti-tumor effects by alleviating immune suppression, and have demonstrated broad applicability in the treatment of solid tumors (Sharma et al., 2021). In clinical practice, it has been observed that immune-related adverse events (irAEs) caused by ICIs, particularly cutaneous irAEs, occur in approximately 30%–50% of patients (Drilon et al., 2016). These cutaneous events present with diverse clinical manifestations, including maculopapular rash, pruritus, bullous dermatitis, Stevens-Johnson syndrome (SJS), toxic epidermal necrolysis (TEN), and reactive cutaneous capillary endothelial proliferation (RCCEP) (Watanabe and Yamaguchi, 2023; Yamaguchi et al., 2023). Clinical research data indicate that severe adverse reactions graded as level 3 or higher (according to the CTCAE v5.0 criteria) may cause significant physical harm to patients or lead to permanent discontinuation of ICIs (Alexandris et al., 2022; Blum et al., 2023). Current studies on cutaneous irAEs are often limited to case reports from clinical trials or investigations with strict inclusion and exclusion criteria, small sample sizes, and short follow-up periods, which restrict the comprehensive assessment of cutaneous irAEs (Blum et al., 2023). Therefore, there is an urgent need for more post-marketing pharmacovigilance data to better understand the toxicity profile and associated factors of cutaneous irAEs. This study aims to explore the population stratification, toxicity spectrum, and associated factors of cutaneous irAEs by mining and analyzing cases from the FDA Adverse Event Reporting System (FAERS) database, in order to provide reference for safe clinical use of ICIs.

## 2 Materials and methods

### 2.1 Materials

For this research, the data utilized was derived from the comprehensive original FAERS database, which is accessible via the U.S. Food and Drug Administration’s official website (FDA, 2024-09). The FAERS database has been publicly accessible to researchers and healthcare professionals since its inception in the early months of 2004, with data continuously updated and released on a quarterly basis. In this research, we analyzed the unprocessed ASCII data sets spanning the initial four-quarters of 2004 through the last quarter of 2024 (from 1 January 2004 to 31 December 2024). The raw records was retrieved and transferred into SAS 9.4 software for subsequent data processing and statistical evaluation.

### 2.2 Data processing and analysis

#### 2.2.1 Data cleaning

First, redundant data submissions were systematically eliminated as per the FDA’s guidance in reference (Sakaeda et al., 2013). To ensure clarity, the DEMO table’s PRIMARYID, CASEID, and FDA_DT fields were chosen for analysis and then organized in a specific sequence. The selection process involved sorting these values first by CASEID, followed by FDA_DT, and finally PRIMARYID. When multiple reports share the same CASEID, the report that was most recently updated based on the FDA_DT timestamp will be preserved. When both the CASEID and FDA_DT values are exactly the same, the record containing the highest PRIMARYID number will be retained (Yu et al., 2021).

#### 2.2.2 Data standardization

In the context of immune checkpoint inhibitor-induced cutaneous adverse events, the data items were systematically categorized and assigned standardized labels drawn from the Medical Dictionary for Regulatory Activities (MedDRA Version 27.1) to ensure consistency in classification. The drug nomenclature was systematically standardized in accordance with the WHO Drug Dictionary, which was updated to its September 2024 version. In this research, we have harmonized and combined the latest Social Capital (SOC) categorization model with the established framework for defining Power Transaction (PT), thereby creating a unified system that seamlessly merges these two critical components. This approach not only maintained the relevance of the terminology throughout the analysis process but also preserved the uniformity of the classification framework, thereby reinforcing the integrity of the system’s logical structure (Xiong et al., 2023; Shu et al., 2024).

#### 2.2.3 Data filtering

A total of 22,375,298 patient reports were obtained from the FAERS database in this study. After removing duplicate reports according to the FDA’s rules, 3,761,306 duplicate reports from the same patients were removed, leaving 18,613,992 unique patient reports. These reports included 55,357,463 adverse event reports (a single patient report may contain multiple different adverse events). Further filtering of the data resulted in the inclusion of 55,357,463 reports from 18,613,992 patients for analysis, among which 170,008 reports were related to ICIs. These included 446,680 reports of adverse drug events (ADEs). The target ADEs were identified using the preferred terms in the MedDRA dictionary. A total of 13,223 patients with the target ADEs were identified, involving 15,768 reports of the target ADEs.

### 2.3 Statistical analysis

Signal detection was performed simultaneously with three disproportionality methods: Reporting Odds Ratio (ROR), Bayesian Confidence Propagation Neural Network (BCPNN), and proportional reporting ratio (PRR). ROR identified a potential signal when at least three spontaneous reports were available and the lower 95% confidence limit exceeded 1, with larger values reflecting stronger evidence. BCPNN used the Information Component (IC); a signal was triggered when IC–2 SD (SD = standard deviation) rose above zero. PRR, noted for higher specificity than ROR, applied the thresholds as a ≥3 and the lower limit of the 95% CI > 1 (Shu et al., 2024). Correlation analysis was conducted using both univariate and multivariate logistic regression analyses to explore independent risk factors associated with the occurrence of irAE-Cutaneous. A P value less than 0.05 was considered statistically significant. Heatmaps for PT signal value were clustered using Pearson correlation and average linkage; case-number heatmaps were with Euclidean distance and complete linkage.

## 3 Results

### 3.1 Basic information of patients with target ADEs

**FIGURE 1:**
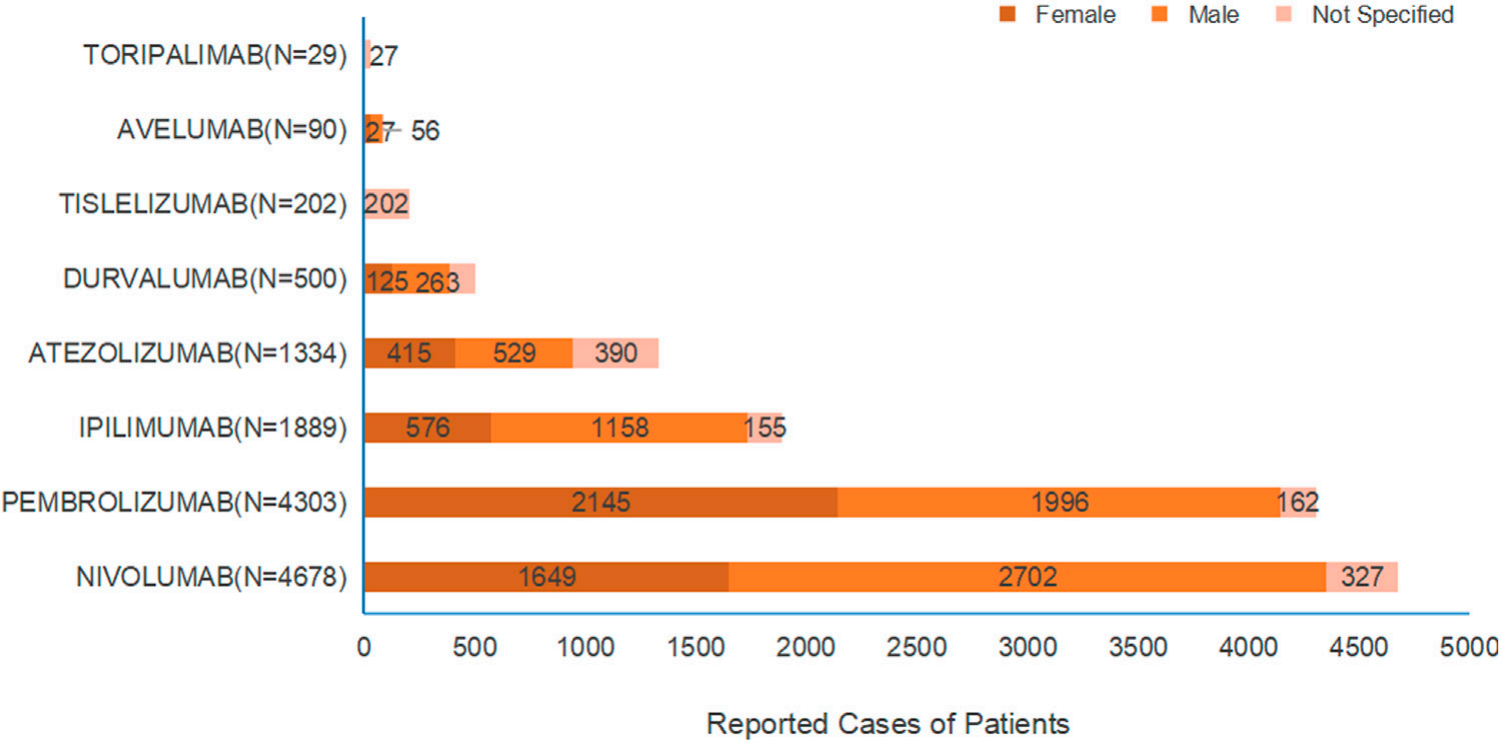
Gender Distribution of Patients with Target ADE Reports [Figure legend: Each drug is grouped by male, female, and not specified. Brown indicates male, orange indicates female, and pink indicates “not specified”].

As shown in Figure 1, among the 13,223 patients who experienced the target ADEs, there were 4,937 female patients (37.9%) and 6,706 male patients (51.49%). When examining the different drugs, the number of female patients exceeded that of male patients only for pembrolizumab, while for the other drugs, the number of male patients was higher than that of female patients.

**FIGURE 2:**
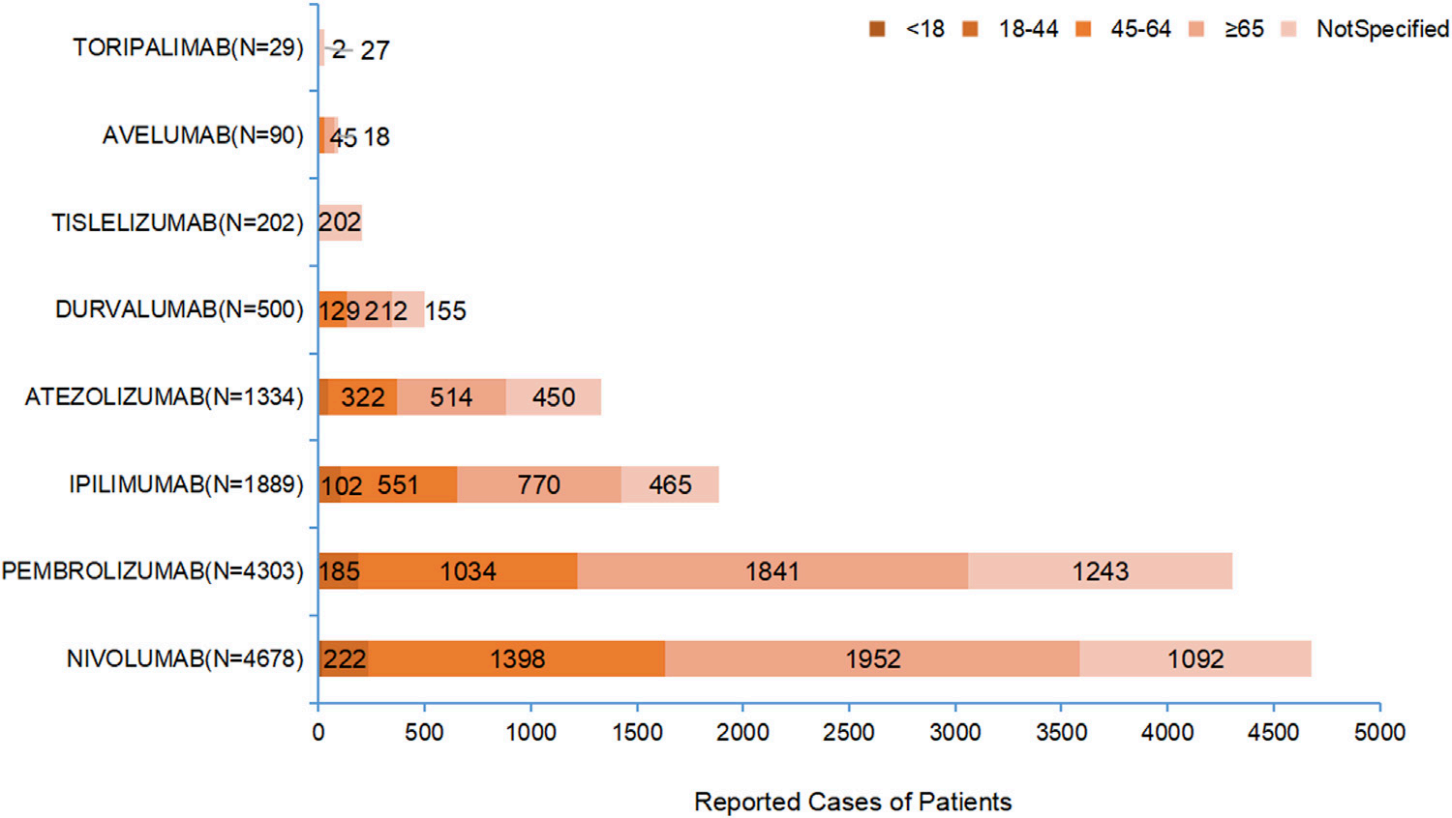
Age Distribution of Patients with Target ADE Reports. [Each drug is grouped by the following age bands: <18, 18–44, 45–64, ≥65, and not specified. Prominently mark the counts in the 45–64 and ≥65 segments].

Among the patients who reported the target ADEs, the majority were aged over 65 years, accounting for 5,336 cases (40.97%), followed by patients aged 45–64 years, who accounted for 3,459 cases (26.56%). As shown in Figure 2, for all the drugs, the primary population experiencing irAE-Cutaneous was patients aged over 65 years, with a significantly higher number than other age groups.

### 3.2 Drug signal detection results

**FIGURE 3:**
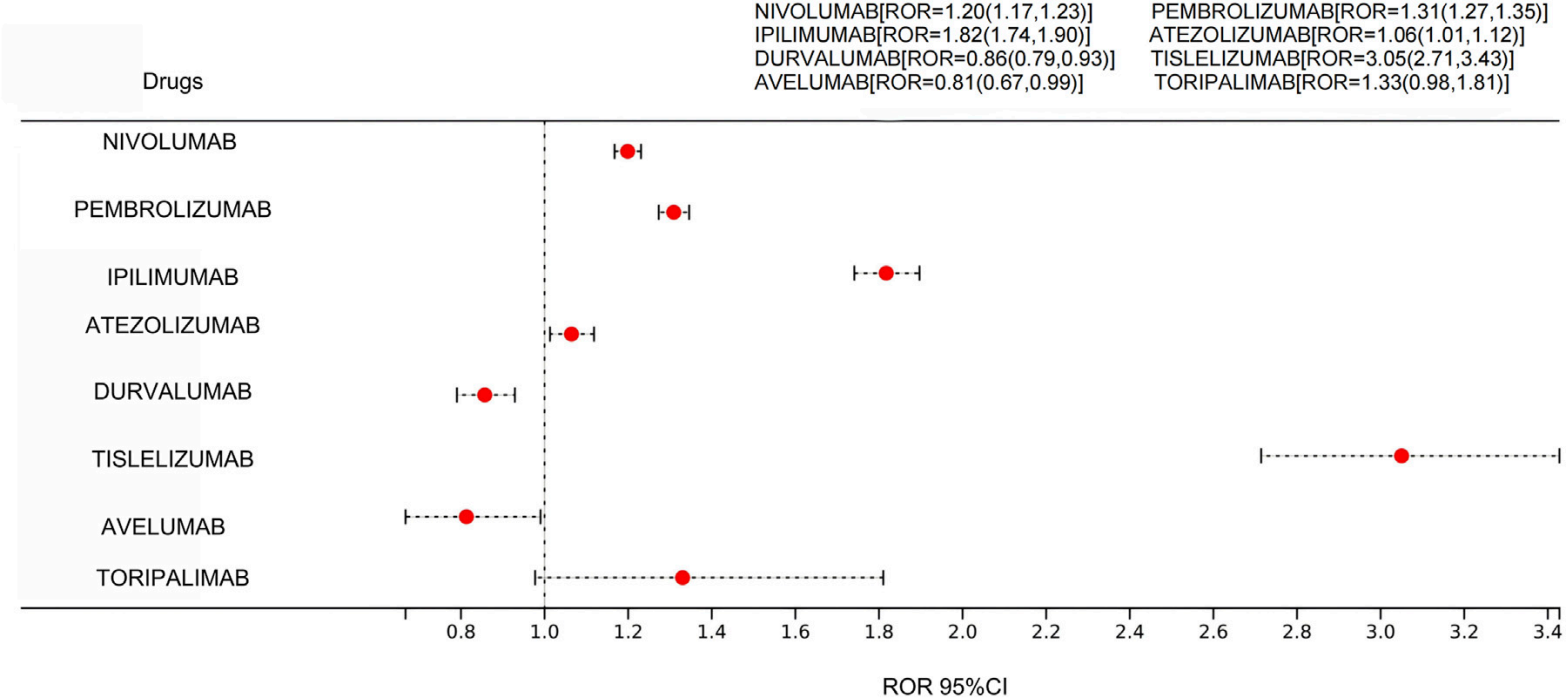
Forest Plot of Drug Signal Detection Results. [Forest plot of the reporting odds ratio (ROR) with 95% confidence interval. If a horizontal line does not cross the vertical reference (ROR = 1), the drug–adverse-event pair shows a statistically significant signal; lines lying entirely to the right indicate a positive ROR signal, and the farther right the line, the stronger the signal].

As shown in Figure 3, the target ADEs reported involved eight “primary suspect drugs.” Five of these drugs produced positive signals using the ROR method, while three drugs had reports of the target events but did not meet the threshold for positive signals, and the details are presented in the forest figure below.

To minimize the potential bias of a single algorithm, this study employed another two methods for detection simultaneously. The results were consistent with those obtained using the ROR method alone. The drugs that tested positive for a signal were the same: nivolumab, pembrolizumab, ipilimumab, atezolizumab, and tislelizumab, as shown in Table 1.

**TABLE 1:** Results of BCPNN and PRR detection.

Drug name	Case	IC	IC025	PRR (95% CI)	Chi-Square
NIVOLUMAB	5725	0.25	0.21	1.19 (1.16,1.22)	181.56
PEMBROLIZUMAB	5168	0.37	0.33	1.30 (1.26,1.33)	362.15
IPILIMUMAB	2201	0.83	0.76	1.78 (1.71,1.85)	767.71
ATEZOLIZUMAB	1620	0.09	0.01	1.06 (1.01,1.11)	6.12
DURVALUMAB	604	−0.22	−0.35	0.86 (0.80,0.93)	14.08
TISLELIZUMAB	307	1.52	1.34	2.88 (2.59,3.21)	388.79
AVELUMAB	101	−0.29	−0.62	0.82 (0.67,0.99)	4.24
TORIPALIMAB	42	0.39	−0.12	1.32 (0.98,1.77)	3.32

### 3.3 Signal intensity of adverse events

**FIGURE 4:**
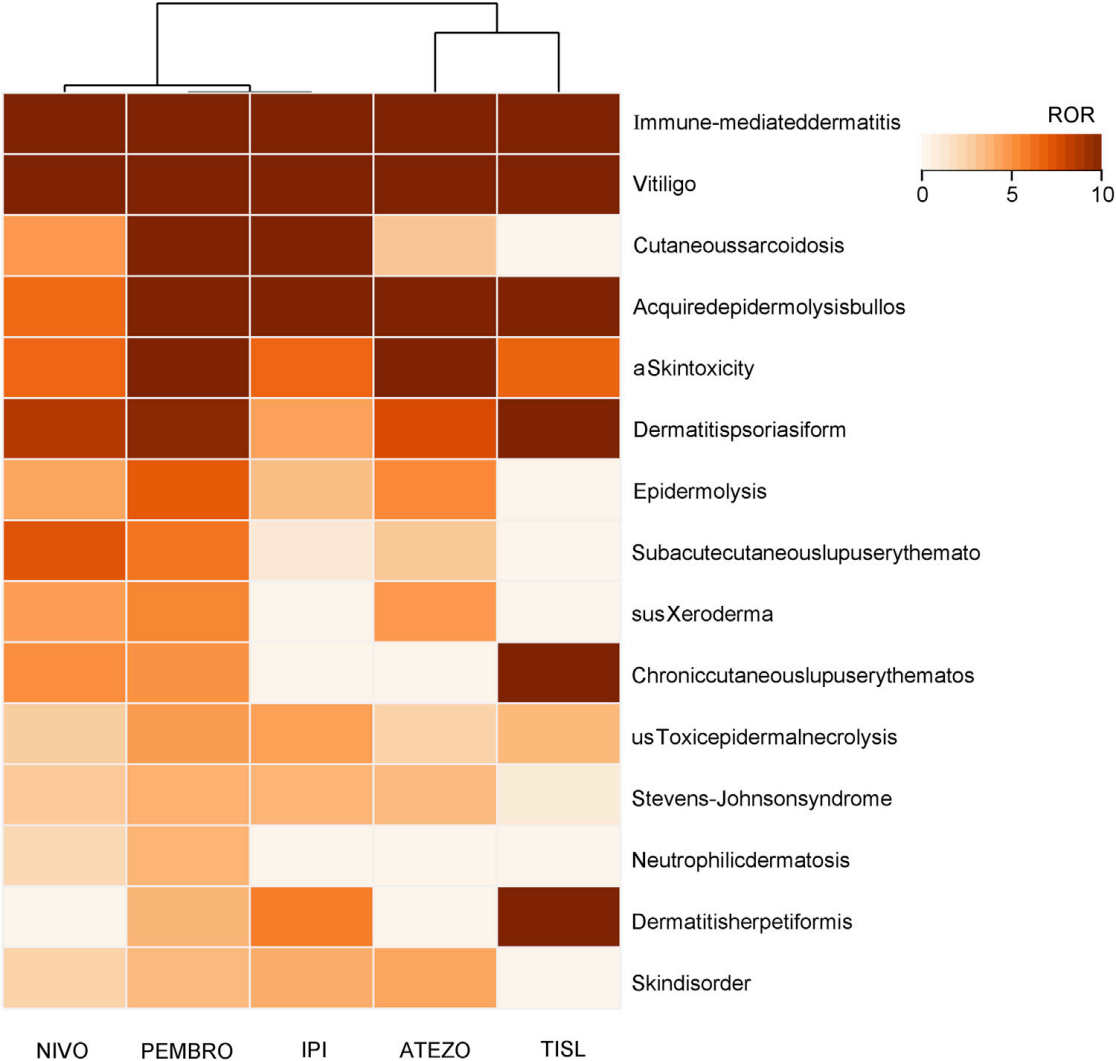
Heat Map for PT Signal Intensity Distribution of Positive Drugs. [Colors represent ROR values; deeper color indicates higher signal intensity. Clustering was performed on drugs (columns) only (Pearson, average linkage); adjacent columns indicate drugs with the closest reporting spectrum. Only the top 15 adverse reactions ranked by ROR value are displayed. NIVO:Nivolumab; PEMBRO:Pembrolizumab; IPI:Ipilimumab; ATEZO:Atezolizumab; TISL:Tislelizumab].

This study further analyzed the signal intensity of cutaneous adverse reactions associated with the five drugs mentioned above. A total of 52 irAE-Cutaneous events exhibited positive signals of varying strengths, and top 15 with the highest signalas were shown in Table 2. Among all the adverse reactions, immune-mediated dermatitis had a strong signal, arranged by signal strength: Ipilimumab > pembrolizumab > tislelizumab > nivolumab > atezolizumab. The adverse reactions with strong signal intensity across several drugs included: immune-mediated dermatitis, vitiligo, cutaneous sarcoidosis, acquired epidermolysis bullosa, skin toxicity, dermatitis psoriasiform, epidermolysis, subacute cutaneous lupus erythematosus, xeroderma, chronic cutaneous lupus erythematosus, toxic epidermal necrolysis, Stevens-Johnson syndrome, etc. A heatmap of cutaneous adverse reactions with positive signal thresholds was created based on signal intensity, as shown in Figure 4. In the heatmap, nivolumab, pembrolizumab and ipilimumac formed one cluster, with pembrolizumab and ipilimumab as a tighter sub-cluster, whereas atezolizumab and tislelizumab were grouped at a greater distance.

**TABLE 2:** PT signal intensity distribution for target ADE of positive drugs.

PT \ Drug	NIVO	PEMBRO	IPI	ATEZO	TISL
Immune-mediated dermatitis	73.74	219.88	331.51	17.70	83.67
Vitiligo	27.85	28.86	45.53	15.13	15.29
Cutaneous sarcoidosis	4.48	13.24	17.42	2.80	N
Acquired epidermolysis bullosa	6.25	11.42	12.03	19.74	140.18
Skin toxicity	6.33	10.22	6.33	14.69	6.39
Dermatitis psoriasiform	8.38	9.66	4.15	7.34	16.09
Epidermolysis	3.99	6.78	3.07	5.01	N
Subacute cutaneous lupus erythematosus	6.97	5.75	0.83	2.70	N
Xeroderma	4.27	5.16	N	4.46	N
Chronic cutaneous lupus erythematosus	4.89	4.71	N	N	43.78
Toxic epidermal necrolysis	2.52	4.38	4.26	2.15	3.30
Stevens-Johnson syndrome	2.55	3.59	3.45	3.26	0.72
Neutrophilic dermatosis	1.92	3.49	N	N	N
Dermatitis herpetiformis	N	3.42	5.49	N	63.94
Skin disorder	2.23	3.23	3.75	4.02	N

(Due to space constraints, only the top 15 adverse reactions with the highest PT, signal are displayed; “N” denotes a negative signal. NIVO:Nivolumab; PEMBRO:Pembrolizumab; IPI:Ipilimumab; ATEZO:Atezolizumab; TISL:Tislelizumab).

### 3.4 Counts of detected adverse events

**FIGURE 5:**
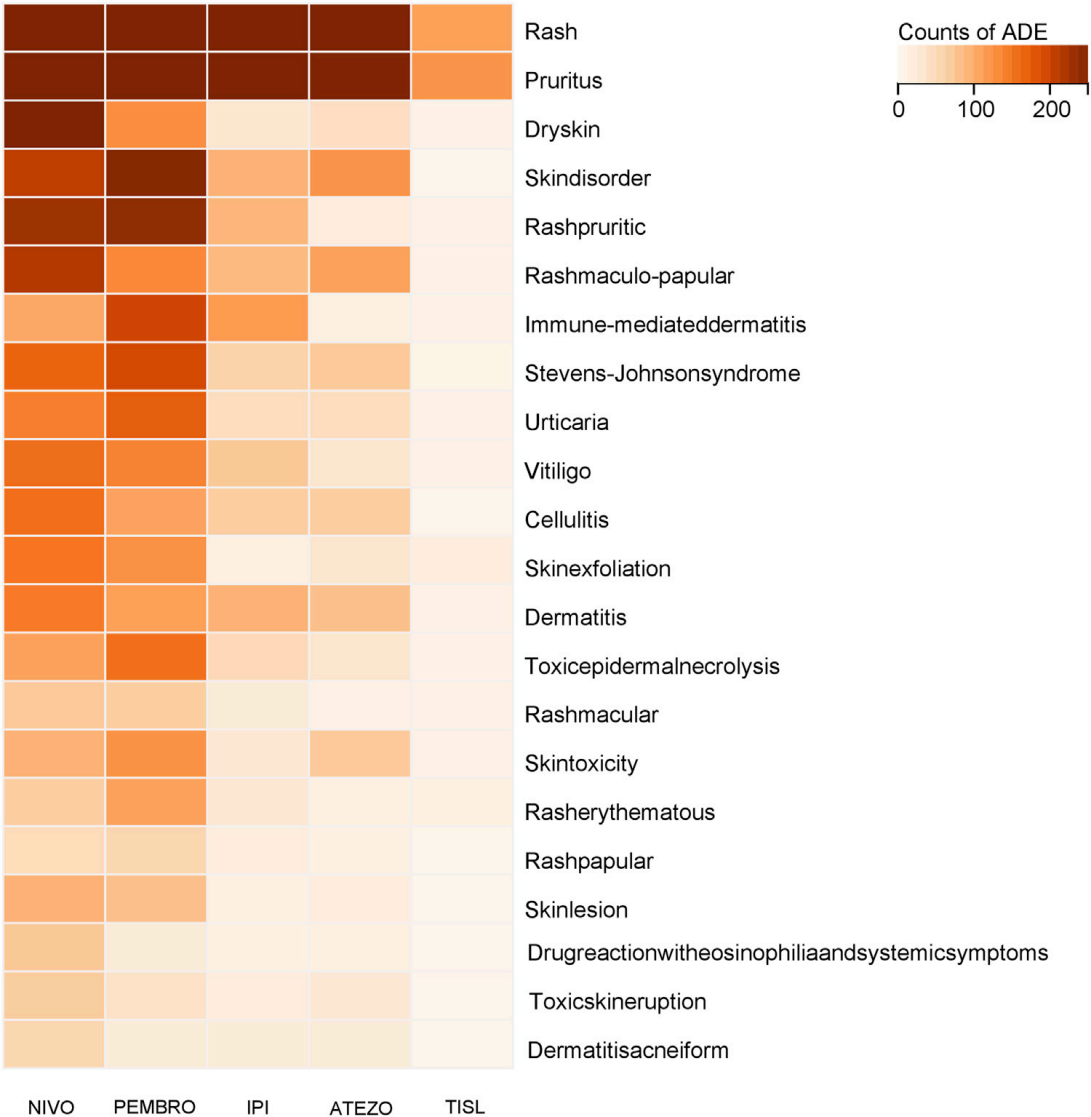
Detection Counts For Target ADE of Positive Drugs. [Heatmap of case counts for immune-mediated skin adverse events, with Euclidean distance and complete linkage method; deeper color indicates higher case numbers; only reactions with >50 cases are shown. NIVO:Nivolumab; PEMBRO:Pembrolizumab; IPI:Ipilimumab; ATEZO:Atezolizumab; TISL:Tislelizumab].

Further analysis of the detection counts of irAE-Cutaneous events (as shown in Figure 5) revealed that rash had the highest number of detected cases. Among these, nivolumab had the largest reported cases (1,739), followed by pembrolizumab (1,710). Pruritus ranked second in terms of detected cases, with the majority of reports coming from nivolumab (1,123 cases) and pembrolizumab (818 cases). Dermatitis bullous was most frequently detected in association with nivolumab (48 cases), pembrolizumab (34 cases), and atezolizumab (25 cases). Serious skin-related adverse reactions, including Stevens-Johnson syndrome (SJS) and toxic epidermal necrolysis (TEN), were also found. When it comes to SJS, pembrolizumab was associated with the highest number of detected cases (187), with nivolumab coming next at 160 cases and atezolizumab at 65 cases. In the case of TEN, the three drugs with the most detected cases were pembrolizumab (148 cases), nivolumab (103 cases), and ipilimumab (45 cases). The main adverse reactions caused by tislelizumab were pruritus and rash. The number of such cases was significantly lower than that of the other four drugs. Moreover, there were very few cases of severe skin adverse reactions, totaling less than 10 cases.

### 3.5 Correlation with other immune-related adverse

The study further analyzed the correlation between immune-mediated dermatitis and other immune-related adverse reactions. Univariate analysis indicated that the P-values for the co-occurrence of pneumonia, hyperthyroidism, hypothyroidism, myositis, and fatigue were all less than 0.05. Logistic regression analysis revealed that, except for pneumonia, the 95% confidence intervals (CI) for the other immune-related adverse reactions did not include 1, and their P-values were all less than 0.05. Among these, patients with fatigue had the highest odds ratio (OR), as shown in Table 3.

**TABLE 3:** Correlation with other Adverse Immune Reactions.

Variable	Univariate		Multivariate				
	Wald x^2^	P-value	Wald x^2^	P-value	β	SE	OR (95%CI)
Pneumonia in combination	7.97	0.004744928	0.05	0.83	0.01	0.06	1.01 (0.898–1.143)
Hyperthyroidism in combination	211.97	5.09242E-48	39.56	<0.0001	0.47	0.07	1.60 (1.382–1.853)
Myositis in combination	226.07	4.2879E-51	35.23	<0.0001	0.33	0.06	1.39 (1.249–1.554)
Fatigue in combination	2502.64	0	1358.86	<0.0001	1.38	0.04	3.99 (3.707–4.294)
Hypothyroidism in combination	529.52	3.5797E-117	110.48	<0.0001	0.55	0.05	1.73 (1.565–1.922)

## 4 Discussion

ICIs have achieved extending survival of patients with advanced solid tumors. However, as the primary target organ of immune responses (Finn et al., 2020), skin present high incidence of related adverse reactions, ranging from self-limiting maculopapular eruptions to life-threatening Stevens-Johnson syndrome (SJS), which shows significant heterogeneity. Immune-related adverse events (irAEs) relating to those treatments are gradually becoming the bottleneck that limits their clinical application. Therefore, the establishment of a hierarchical management system for skin toxicity reactions and the exploration of individualized intervention strategies have become important directions in current clinical research. This study, based on FAERS database between 2004 and 2024, systematically classified different irAE-Cutaneous to conduct signal detection analysis, aming to summarize the population characteristics of the patients, and explore the differences in the toxicity spectrum of various types of irAE-Cutaneous, and investigate factors associating with the occurrence of irAE-Cutaneous; providing important evidence-based support for optimizing clinical monitoring protocols for ICIs and establishing toxicity early warning models.

### 4.1 Analysis of population characteristics

Analysis based on the large sample data from FAERS indicates that, except for pembrolizumab where females are slightly more than males, all other drugs show a higher proportion of male patients than female patients. In terms of age, the proportion of patients aged 65 and above is the highest for all drugs. Therefore, the occurrence of irAE-Cutaneous exhibits significant demographic characteristics: that is, males and the elderly population aged 65 and above constitute the main affected group. The gender differences shown in this study are consistent with the results of a previous retrospective study. Fan (2023) conducted a statistical analysis of 1,969 patients who experienced ADEs after using immune checkpoint inhibitors between 2019 and 2021 and found that the incidence of drug adverse reactions in male patients was higher than that in female patients. This gender difference may be significantly related to lifestyle factors such as smoking and drinking in the male population. Tobacco and ethanol can alter pharmacokinetics and drug bioavailability, thereby increasing the risk of target organ toxic reactions (Smith, 2009). Additionally, tobacco and alcohol are important factors that cause changes in autoimmune reactions, and the imbalance of immune homeostasis may be an important reason for the increased incidence of immune-related adverse events (Mazzaschi et al., 2023). The results of the data mining show that the incidence of cutaneous toxicity in patients aged 65 and above is higher than that in younger groups, which is consistent with the findings of a retrospective studies. Multiple clinical trials and real-world studies have shown that elderly patients may have a higher risk of developing s-irAEs, and the incidence of grade ≥3 s-irAEs is also higher in this population compared to the general population (Reck et al., 2019; Jassem et al., 2021). It has been found that the number of skin dendritic cells (DCs) decreases in an age-dependent manner, accompanied by reduced expression of key molecules in the TLR signaling pathway (Bhandari et al., 2018). This leads to impaired phagocytic function, dysregulation of lymphocyte co-stimulatory molecules, and consequently, a decline in the skin immune barrier function and an increase in inflammation.

### 4.2 Cutaneous toxicity profiles of various immune checkpoint inhibitors

In this study, three methods—ROR, BCPNN, and PRR—were utilize to systematically assessing the correlation between different immune checkpoint inhibitors and irAE-Cutaneous. The signal strength regards as a quantitative measure of the correlation between a drug and a adverse event, directly reflecting the potential risk of the drug inducing a certain side effect. Five drugs in this study manifested positive signals, including the CTLA-4 inhibitor ipilimumab, the PD-L1 inhibitor atezolizumab, and the PD-1 inhibitors nivolumab, pembrolizumab, and tislelizumab. In the heat-map clustering, ipilimumab and pembrolizumab grouped closely together, implying a largely overlapping cutaneous toxicity profile. Atezolizumab and tislelizumab, however, lay on a more distant branch, reflecting a clearly broader and more heterogeneous pattern of skin immune-related adverse-event reporting.

#### 4.2.1 General cutaneous adverse reaction toxicity profile

The data mining results indicate that the CTLA-4 inhibitor ipilimumab is most strongly associated with immune-mediated dermatitis, followed by the PD-1 inhibitors pembrolizumab and tislelizumab, while the PD-L1 inhibitor atezolizumab has the weakest association. This suggests a high correlation between ipilimumab and the occurrence of this type of cutaneous adverse event. Research has shown that ipilimumab, by specifically binding to the CTLA-4 receptor and blocking the CD80/CD86 co-stimulatory pathway, significantly enhances the activation of antigen-presenting cells, thereby triggering a robust T-cell immune response. This pathological mechanism may account for its high signal intensity in immune-mediated dermatitis. Immune-mediated dermatitis is a type of skin inflammation characterized by immune dysregulation (Diotallevi et al., 2022), with common features including chronic inflammatory reactions mediated by T cells and antigen-antibody interactions, encompassing conditions such as psoriasis, atopic dermatitis, alopecia areata, and vitiligo. Studies have confirmed that ipilimumab can activate CD8^+^ T cells and inhibit the expression of melanoma-related antibodies, thereby inducing vitiligo (Weber et al., 2015). Therefore, the CTLA-4 inhibitor ipilimumab exhibits an extremely strong signal for vitiligo (ROR value = 45.53), significantly higher than that of nivolumab and pembrolizumab. In contrast, the PD-1 inhibitors nivolumab and pembrolizumab show higher signals for psoriasis, indicating a significant correlation between these two drugs and the occurrence of psoriasis. The possible mechanism lies in the fact that PD-1 inhibitors can significantly regulate the imbalance between Th1 and Th17 cells, promoting the recruitment of pro-inflammatory cytokines such as neutrophils and the proliferation of epidermal keratinocytes, thereby leading to the deterioration of psoriasis or inducing it (Watanabe and Yamaguchi, 2023). Tislelizumab demonstrates a high signal for exfoliative dermatitis (ROR value = 21.2) and a significant association with bullous dermatitis (ROR value = 9.08), higher than other drugs, suggesting that these adverse reactions should be particularly monitored with this medication. For adverse reactions such as pruritus, pruritic rash, papules, acneiform eruptions, and pustules, weak associations were observed with all drugs, indicating that these reactions are generally widespread and common, without significant correlation with specific drugs.

Although the ICIs examined in this study showed weak associations with rash and pruritus, the cases for these two events greatly exceeded those for other adverse reactions. This further supports the prevalence of rash and pruritus, regardless of whether they were CTLA-4 inhibitors, PD-1 inhibitors, or PD-L1 inhibitors. Clearly, an increase in absolute case numbers does not necessarily reflect a higher correlation.

For irAE-Cutaneous highly associated with specific drugs (such as vitiligo and psoriasis), there is also a bias between case numbers and signal strength. Taking the incidence of vitiligo, for example, the CTLA-4 inhibitor ipilimumab has a stronger positive signal than PD-1 inhibitors. However, the number of cases reported from ipilimumab is 67, while nivolumab and pembrolizumab is 152 and 132 respectively. This contradiction may stem from exposure bias. Clinically, PD-1 inhibitors have been approved for a wider range of indications, and thus are used more frequently, leading to an increased incidence of adverse reactions. In contrast, the application of CTLA-4 inhibitors is mainly focused on specific cancer types such as melanoma. In this study, the total case number of PD-1 inhibitors (nivolumab + pembrolizumab) was 10,893 cases, which is 4.95 times that of the CTLA-4 inhibitor group (ipilimumab with 2,201 cases). Therefore, signal strength better reflect the “specific association strength” between a drug and an event; While absolute case numbers are directly affected by the size of the exposed population and are related to the prevalence of drug use, which maybe require standardized incidence ratio (SIR) before evaluating the number of occurrences to minimized this deviation.

#### 4.2.2 Toxicity profile of severe cutaneous adverse reactions

From a clinical perspective, with the widespread use of immune checkpoint inhibitors, the management of fatal skin toxicity events related to ICIs has become significantly more challenging. According to the WHO Drug Safety Alert classification criteria (Goldinger et al., 2016), Stevens-Johnson syndrome (SJS) and toxic epidermal necrolysis (TEN), as the severe manifestations of immune-related skin toxicity, have a core pathological mechanism of extensive peeling of the skin and mucous membranes due to rapid apoptosis of epidermal cells. Severe patients may also develop sepsis (with a 30% in-hospital mortality rate), ARDS, and MODS and other systemic damages (Bhardwaj et al., 2022; Okiyama and Tanaka, 2022). The exact pathogenesis of TEN has not been fully elucidated, but its core mechanism involves abnormal regulation of the PD-1/PD-L1 signaling pathway, leading to immune homeostasis imbalance. Some studies suggest that ICIs can trigger skin reactions through type IV hypersensitivity. Under normal physiological conditions, PD-L1 is not typically detectable in epidermal keratinocytes. However, ICIs can trigger excessive activation of T cells through the PD-1/PD-L1 signaling pathway, disrupting peripheral immune tolerance and leading to abnormal activation and clonal expansion of autoreactive CD8^+^ T lymphocytes, inducing a cascade of apoptosis in keratinocytes. At the same time, dysfunction of Tregs and the enhancement of co-stimulatory factors exacerbate local inflammatory responses, ultimately resulting in extensive necrosis and detachment of the epidermis (Vivar et al., 2017; Chen et al., 2018).

The results of this study reveal that all five drugs exhibited positive signals for SJS/TEN, with statistical significance. Among them, the PD-1 inhibitor pembrolizumab showed a stronger correlation with SJS/TEN compared to other drugs. The CTLA-4 inhibitor ipilimumab ranked second in terms of risk for SJS/TEN occurrence. The PD-L1 inhibitor atezolizumab had the weakest association with TEN among the five drugs, while tislelizumab had a lower correlation with SJS compared to the other drugs. These findings are consistent with a meta-analysis that included 20 RCTs from the United States, the United Kingdom, Italy, and France, involving 3,813 patients (Zihang et al., 2022). Pembrolizumab was associated with the most severe cutaneous adverse reactions, and high-dose ipilimumab or combination therapy with nivolumab and ipilimumab also had higher incidence rates of cutaneous adverse reactions.

In this study, tislelizumab had 1 report of SJS and 3 reports of TEN. However, since the overall number of cutaneous adverse reaction reports for this drug was significantly lower than that for other drugs, further clinical data accumulation is needed to confirm its exact safety profile and toxicity spectrum.

### 4.3 Correlation with other immune-related adverse reactions

This study investigated the correlation between irAE-Cutaneous and other common immune-related adverse reactions. The results of univariate analysis showed that the P-values for variables such as concurrent pneumonia, hyperthyroidism, myositis, fatigue, and hypothyroidism were all significantly lower than 0.05, indicating a significant association with the study objective. In the multivariate analysis, except for concurrent pneumonia (P = 0.83), which had a P-value greater than 0.05 and thus was not significantly associated with the study objective, the other variables (hyperthyroidism, myositis, fatigue, and hypothyroidism) all had P-values lower than 0.05. This means that after excluding the interference of other factors, concurrent pneumonia did not significantly affect the occurrence of cutaneous adverse reactions, while hyperthyroidism, myositis, fatigue, and hypothyroidism still had significant impacts.

Further in-depth analysis combined with literature review reveals that the impact mechanism of thyroid hormones on immune-mediated dermatitis is relatively well-defined. Thyroid hormones can regulate the proliferation, differentiation, and activity of immune cells through the T3 receptors in the skin. When thyroid hormone levels are abnormal, it not only damages the structure and function of the dermis but also disrupts the normal function of T cells such as CD4^+^ or CD8^+^, prompting the immune system to attack abnormal antigens in the epidermis and dermis, destroy the skin barrier, and thus trigger immune-mediated dermatitis (He et al., 2020; Zhou et al., 2018).

Immune-related myositis is characterized by the immune system targeting muscle tissue. During this process, immune system generate autoantibodies that against the antigens in both muscle and skin and trigger the immune cross-reactivity, which leads to the release of a substantial amount of pro-inflammatory cytokines, and ultimately induces inflammatory responses in the skin and causes immune-related dermatitis (Zhu, 2022). Clinical studies (Kostine et al., 2018; Kostine et al., 2019) have also confirmed the association between these two adverse events. A single-center prospective study in France indicated that approximately 45% of patients with immune-related myositis also experienced immune-related skin toxicity problem. Similarly, A multicenter study in Canada also indicates that approximately half of patients with immune-related myositis will have skin adverse reactions, with the main clinical syndrome as muscle weakness or muscle pain.

Cancer-related fatigue involves multiple aspects of neuroendocrine-immune regulation (Bower and Lamkin, 2013). When patients are in a state of fatigue, the immune system’s surveillance and defense capabilities are diminished, stress hormone secretion is disrupted, and the activity of immune cells and cytokine secretion are affected. The immune system’s ability to recognize and regulate its own tissues becomes abnormal, which is very likely a key factor in triggering immune-mediated dermatitis.

In summary, based on the above studies and analyses, patients with concurrent thyroid hormone abnormalities, myositis, and fatigue have a significantly increased risk of developing immune-related cutaneous adverse reactions.

### 4.4 Limitations

This study, based on pharmacovigilance analysis using a spontaneous reporting system, needs to address the following limitations: First, regarding data quality, the few proportion of “not specified” entries for sex, age, and race in the FAERS database undermines the reliability of demographic stratifications; meanwhile, systematic biases may arise from duplicate reports, under- or over-reporting, temporal recording discrepancies, and misspellings of drug names. Second, in terms of statistical dimensions, the lack of a denominator for the exposed population means that true incidence rates cannot be calculated. Finally, the risk signals only reflect the statistical association between drugs and adverse events (AEs), and their causality needs to be verified through active surveillance methods such as prospective cohort studies.

## 5 Conclusion

This study, based on pharmacovigilance analysis using the FAERS, has revealed the heterogeneous characteristics of the cutaneous toxicity profiles associated with ICIs, and the specific toxic spectrum of each ICIs; also identified the association between the other irAEs and irAE-Cutaneous. The findings hold significant value for guiding clinical practice. The evidence presented suggest that an individualized AE monitoring system needs to be established in clinical practice, and a tiered management approach should be implemented in clinical applications: Conduct baseline screening for high-risk populations such as the elderly and those with a history of autoimmune diseases to avoid the use of drugs with corresponding toxicity profiles. Use a dynamic dermatological scoring system during treatment to monitor early symptoms, and improve the safety threshold of cancer immunotherapy through early identification and precise intervention. For patients who have developed ICIs - related hyperthyroidism, myositis, fatigue, or hypothyroidism, attention should be paid to the occurrence of severe irAE-Cutaneous.

## Data Availability

The original contributions presented in the study are included in the article/Supplementary Material, further inquiries can be directed to the corresponding author.
