# Supplementary material for: Spectrum mining of immune checkpoint inhibitor-related cutaneous toxicities and analysis of associated factors based on FAERS
Source: Front Pharmacol. 2026 Jan 2;16:1684390. doi: 10.3389/fphar.2025.1684390 (PMC12808339; doi:10.3389/fphar.2025.1684390)
Supplement: Supplementary file 1 [file Table2.docx]

# Supplementary Material

**Table 2: PT Signal Distribution of target ADE for Each Drug**

| PT \ Drug | NIVOLUMAB | PEMBROLIZUMAB | IPILIMUMAB | ATEZOLIZUMAB | TISLELIZUMAB |
| --- | --- | --- | --- | --- | --- |
| Immune-mediated dermatitis | 73.737 | 219.877 | 331.505 | 17.698 | 83.666 |
| Vitiligo | 27.852 | 28.855 | 45.531 | 15.131 | 15.285 |
| Cutaneous sarcoidosis | 4.483 | 13.239 | 17.419 | 2.8 | N |
| Acquired epidermolysis bullosa | 6.25 | 11.421 | 12.025 | 19.739 | 140.175 |
| Skin toxicity | 6.326 | 10.217 | 6.333 | 14.693 | 6.388 |
| Dermatitis psoriasiform | 8.376 | 9.656 | 4.151 | 7.339 | 16.095 |
| Epidermolysis | 3.993 | 6.779 | 3.073 | 5.008 | N |
| Subacute cutaneous lupus erythematosus | 6.974 | 5.746 | 0.829 | 2.702 | N |
| Xeroderma | 4.272 | 5.155 | N | 4.458 | N |
| Chronic cutaneous lupus erythematosus | 4.892 | 4.707 | N | N | 43.778 |
| Toxic epidermal necrolysis | 2.519 | 4.384 | 4.259 | 2.15 | 3.298 |
| Stevens-Johnson syndrome | 2.548 | 3.599 | 3.448 | 3.255 | 0.715 |
| Neutrophilic dermatosis | 1.922 | 3.488 | N | N | N |
| Dermatitis herpetiformis | N | 3.421 | 5.485 | N | 63.939 |
| Skin disorder | 2.234 | 3.232 | 3.751 | 4.023 | N |
| Epidermolysis bullosa | N | 3.209 | N | N | 59.993 |
| Rash follicular | N | 2.794 | N | 22.26 | N |
| Rash maculo-papular | 3.519 | 2.524 | 5.153 | 5.399 | 1.493 |
| Exfoliative rash | 1.05 | 2.381 | 0.508 | 0.827 | N |
| Dermatitis | 2.574 | 2.364 | 6.322 | 4.315 | 3.33 |
| Rash morbilliform | 2.394 | 2.243 | 2.055 | 2.089 | 5.982 |
| Cutaneous lupus erythematosus | 1.073 | 2.163 | 0.693 | 2.824 | 8.081 |
| Dermatitis bullous | 2.421 | 2.064 | 2.143 | 3.968 | 9.079 |
| Nodular rash | 1.626 | 1.961 | N | N | N |
| Rash pruritic | 1.539 | 1.957 | 2.264 | 0.302 | 1.548 |
| Rash | 1.401 | 1.665 | 2.728 | 1.298 | 3.884 |
| Toxic skin eruption | 2.239 | 1.41 | 2.06 | 2.57 | N |
| Seborrhoeic dermatitis | 0.905 | 1.365 | N | 2.142 | N |
| Epidermal necrosis | 0.745 | 1.35 | N | 1.175 | N |
| Dermatitis acneiform | 3.058 | 1.246 | 4.015 | 3.456 | N |
| Skin lesion | 1.183 | 1.2 | 0.364 | 0.508 | N |
| Dermatitis exfoliative generalised | 2.471 | 1.13 | 3.64 | 4.073 | 5.289 |
| Hyperkeratosis | 3.075 | 1.098 | 0.252 | 0.205 | N |
| Rash erythematous | 0.5 | 1.026 | 0.776 | 0.289 | 3.774 |
| Papule | 1.136 | 0.978 | 1.258 | 1.193 | 14.684 |
| Rash papular | 0.671 | 0.961 | 0.787 | 0.295 | N |
| Dermatitis allergic | 0.55 | 0.959 | 0.356 | 0.868 | 2.764 |
| Pruritus | 1.073 | 0.942 | 1.456 | 0.745 | 5.084 |
| Butterfly rash | 0.742 | 0.895 | N | N | N |
| Dermatitis exfoliative | 0.937 | 0.888 | 1.038 | 0.211 | 21.221 |
| Cellulitis | 1.042 | 0.851 | 1.655 | 1.345 | N |
| Rash macular | 0.697 | 0.765 | 0.738 | 0.166 | 2.389 |
| Skin necrosis | 0.756 | 0.746 | 0.8 | 0.433 | 3.107 |
| Skin exfoliation | 0.634 | 0.641 | 0.189 | 0.391 | 2.409 |
| Urticaria | 0.299 | 0.446 | 0.352 | 0.286 | 0.3 |
| Dry skin | 0.799 | 0.414 | 0.286 | 0.331 | 0.385 |
| Dermatitis contact | 0.161 | 0.349 | 0.125 | 0.203 | N |
| Drug reaction with eosinophilia and systemic symptoms | 0.861 | 0.294 | 0.398 | 0.445 | N |
| Skin swelling | 0.24 | 0.173 | N | 0.151 | 2.17 |
| Dermatitis atopic | 0.025 | 0.03 | 0.048 | 0.079 | N |
| Injection site rash | 0.05 | 0.015 | N | 0.039 | N |
| Viral rash | 0.899 | N | 7.006 | 2.839 | N |

（N represents negative signal）
